# Is There Death in the Pot?

**Published:** 1874-04

**Authors:** 


### Editorial.

Is there Death in the Pot?—By the public prints we learn that the trichina spiralis is again making its ravages in the North-West; two victims having lately died of trichiniasis at Aurora, Indiana. A panic is said to exist in that region, and the pork market is reported dull—very dull, whilst the consumption of pork and sausage, for the time being, is practically tabooed.

It is well known that modern organic chemistry with giant strides is treading so closely upon the heels of dame Nature as to evolve from the chemical laboratory a long series of subtile ethers, which, by skillful combinations, are made to resemble the flavor and odor of fruits and flowers so closely as, practically, to drive *nature* completely from the market in flavoring extracts and perfumes. A few years ago the country was permeated in every quarter by peregrinating philosophers and alchemists who, for the paltry consideration of one dollar lawful money, imparted the magical art of transmuting a gallon of milk, weighing about eight pounds, into ten pounds of wholesome butter for the family table.

And now comes the ubiquitous genius of Yankee ingenuity, with "good fresh butter," evolved from beef tallow, with as much facility as nutmegs or cucumber seed are turned out of beech-wood. We quote from *The Druggist* for January:

The real fat being thus separated from the superfluous animal matter, the next step is the separation of the olein from the margarin and stearin. To accomplish this the material is pressed, being first put in cotton cloths, through the pores of which the oil drips into a vessel. It is this oil, olein, containing in solution more or less margarin and stearin from which the butter is to be churned. The residum left in the bags is solid stearin, which is used for candle making. The churns have revolving paddles, and the oil on being placed in the churns is mixed with one-fifth of the weight of sour milk. The churning operation is continued for twenty minutes, when the compound has assumed the semi-solid condition of soft butter, which a slight diminution of temperature renders firm. The churns are worked in a cool chamber, rendered so by means of a reservoir of ice suspended overhead. The butter is now colored yellow by admixture of a little vegetable annatto, which is harmless, and after being salted is worked like ordinary butter, on a working table, with a presser. The churning of the oil with the sour milk increases its weight from absorption of water, so that three pounds of oil will make four pounds of butter. From one hundred pounds of suet seventy pounds of butter are produced, twenty pounds of stearin and ten pounds of scraps.

Although this article can now be made cheaper than butter, it is not believed that it will entirely take the place of the ordinary article. Suet can only be obtained in large quantities in cities; and, as the process is no longer a secret, the price of suet will undoubtedly advance. Even if all the suet obtainable in San Francisco is used in this manner, the quantity of butter manufactured will be insufficient to supply the market. The New York company are now enlarging their works to the capacity of twelve tons of butter per day, but this is only about one-tenth the quantity daily consumed in that city.

The editor of the Rome, Georgia, *Daily Commercial* says of it:

"We have had recently, on our dinner table, some of the new style of butter. It is excellent; cheaper, and goes much further than the ordinary butter. Olein butter is the name," etc.

Years ago, during our pupilage in the analytical laboratory of Prof. Booth of Philadelphia, we were taught to convert starch into dextrine and then into grape-sugar, or glucose, by the chemical action of sulphuric acid. Little did we then dream that the surplus corn of the North-West would be converted into starch, for the transforming agency of the oil-of-vitrol of the East, to produce an artificial *refined syrup* which is now threatening to drive from our markets the product of nature in the sugar-cane. Is it not time to pause in our headlong course, and ponder well the query, "is there death in the pot?"

